# Depression and quality of life in patients with breast cancer undergoing chemotherapy and monoclonal antibodies

**DOI:** 10.3332/ecancer.2019.937

**Published:** 2019-07-10

**Authors:** Francisco Trinca, Paulo Infante, Rui Dinis, Mariana Inácio, Emílio Bravo, Jorge Caravana, Teresa Reis, Sofia Marques

**Affiliations:** 1 Department of Medical Oncology, Hospital do Espírito Santo de Évora EPE, 7000-811 Évora, Portugal; 2 Department of Mathematics/ECT and Center for Research in Mathematics and Applications/IIFA, University of Évora, 7000-671 Évora, Portugal; 3 Department of Surgery, Hospital do Espírito Santo de Évora EPE, 7000-811 Évora, Portugal; 4 Department of Psychiatry and Mental Health, Hospital do Espírito Santo de Évora EPE, 7000-811 Évora, Portugal

**Keywords:** breast neoplasm, quality of life, depression, chemotherapy, antibodies, monoclonal

## Abstract

**Background:**

Depression is one of the major psychiatric morbidities in cancer patients. The purpose of our study was to evaluate the impact of depressive symptoms in the quality of life (QoL) of patients with breast cancer undergoing chemotherapy and monoclonal antibodies treatments.

**Methods:**

Observational, cross-sectional study conducted between April and November 2016. To evaluate the QoL, the EORTC QLQ-C30 and QLQ-BR23 questionnaire were used. The patients were screened for depressive symptoms using the Hospital Anxiety and Depression Scale (HADS-D) and those with a positive HADS-D positive questionnaire were referenced to the Psychiatry and Mental Health Department for further assessment and follow-up.

**Results:**

We included 45 female patients. Sixteen (35.6%) patients had a positive HADS-D questionnaire and depressive symptoms confirmed by a psychiatric physician. Of those patients, 7 (15.6%) had a major depressive episode confirmed by psychiatric interview. There was a significant association of depressive symptoms with the future perspectives scale (*p* = 0.022), breast symptoms scale (*p* = 0.011) and arm symptom scale (*p* = 0.005). Significant differences were found in the fatigue (*p* = 0.024), pain (*p* = 0.037) and dyspnea (*p* = 0.009) subscales being worse in patients with depressive symptoms. The association between having depressive symptoms or not was shown to be significant or marginally significant for the variables stage of the tumour (*p* = 0.057), presence of distant metastasis (*p* = 0.072) and previous diagnosis of depression (*p* = 0.011). The patients treated with regimens containing monoclonal antibodies presented better outcomes in various subscales of the EORTC QLQ-C30 and QLQ-B23 questionnaires than those patients treated with chemotherapy regimens without monoclonal antibodies.

**Conclusions:**

Despite the small sample of our study, this study provided evidence that depressive symptoms in patients with breast cancer undergoing chemotherapy and monoclonal antibodies treatments detrimentally reduced various aspects of QoL.

## Background

Depression is one of the major psychiatric morbidity in cancer patients [1]. Between 25% and 50% have psychological problems [2]. A meta-analysis found that the prevalence of major depressive disorder was 14.9% in those patients [3].

The types of cancer most associated with depression are the cancers of the head and neck (22% to 57%) [4], pancreas (33% to 50%) [5], breast (1.5% to 46%) [6] and lung (11% to 44%) [7].

Even though the mechanism by which psychological distress impacts on the efficacy of breast cancer treatment is not completely understood, there is evidence that suggests that psychological distress may cause stress that alters the hormonal and neuronal secretions affecting the biological activity of breast cancer cells [8].

Multiple factors make it difficult to diagnose mood disorders in cancer patients. The symptomatology of the disease or its treatments often overlaps with the symptoms investigated in the evaluation of depression, such as anorexia, weight loss, insomnia, loss of interest in activities, lack of energy or cognitive deficit [9, 10].

The Hospital Anxiety and Depression Scale (HADS) is one of the most used scales to evaluate symptoms of anxiety and depression because it excludes the somatic symptoms of depression and anxiety, such as fatigue, loss of appetite, pain or insomnia, which can also be caused by cancer or its treatments [11]. It was developed to identify symptoms of anxiety and depression in patients from non-psychiatric hospitals [11], and was subsequently used in outpatients [13] and in healthy persons [14].

The scale consists of 14 items and is composed of two subscales, one for anxiety and the other for depression. Each of the subscales has seven items. Each item is scored from 0 to 3. The total scores for each subscale are calculated by the individual sum of each response of the subscales. A higher score indicates more distress. The cut-off point for anxiety or depression is 8 [15].

In a comparative study with the Diagnostic and Statistical Manual of Mental Disorders, 4th Edition structured interview for major depression in patients admitted on oncology surgical wards. Keller *et al* [12] determined that the sensitivity and specificity of HADS were 86% and 87%, respectively, demonstrating their validity and utility in the screening.

Quality of life (QoL) is an important outcome of the disease and its treatments, and for that, it has been evaluated and measured in the last decades. The EORTC QLQ-C30 questionnaire has shown in a number of studies to be a reliable and valid tool [16]. It consists of 30 questions, which are subdivided into three scales: the global health status and quality of life (QL2); functional scales and the symptomatic scales are composed by fatigue (FA), nausea and vomiting (NV), pain (PA), dyspnea (DY), insomnia (SL), appetite loss (AP), constipation (CO), diarrhoea (DI) and financial difficulties (FI) [17].

The QLQ-BR23 questionnaire is a QLQ-C30 supplemental questionnaire developed specifically for breast cancer patients. It consists of 23 questions, which are subdivided into two scales: the functional scales, composed by body image (BRBI), sexual functioning (BRSEF), sexual enjoyment (BRSEE) and future perspective (BRFU) and the symptom scales, composed by the subscales systemic therapy side effects (BRST), breast symptoms (BRBS), arm symptoms (BRAS) and upset by hair loss (BRHL) [17].

Although some information is available regarding the effects of depression in the QoL of patients with breast cancer undergoing antineoplastic treatments, no data is found about their impact on the Portuguese population with breast cancer and depressive symptoms.

## Methods

Observational, cross-sectional study conducted between April and November 2016 at Hospital do Espírito Santo de Évora in Portugal.

The purpose of our study was to evaluate the impact of depressive symptoms in the QoL of patients with breast cancer undergoing chemotherapy and monoclonal antibodies treatments.

The patients were recruited and invited to join the study before the start of chemotherapy and monoclonal antibodies treatments scheduled in the Unit of Oncology at our hospital.

All the patients completed informed consent and the project was approved by the Ethics Committee of our hospital. The database was anonymous and registered in the Portuguese National Commission of Data Protection.

We included patients with breast cancer undergoing chemotherapy with or without monoclonal antibodies treatments for at least 2 months.

The exclusion criteria were patients with a minimum life expectancy (less than 3 months), under 18-year old or the presence of cerebral metastasis.

For the comparison of the group of patients with depressive symptoms and without depressive symptoms in the various scales of the QLQ-C30, QLQ-BR23 and the Hospital Anxiety and Depression Scale (HADS-D), the *t*-test was used, whenever both samples could be admitted to come from populations with normal distribution (tested with the Shapiro–Wilk test), otherwise the Wilcoxon–Mann–Whitney nonparametric test was used. To evaluate the effect of some clinical variables in the existence, or not, of depressive symptoms, as well as the effect of QoL, BRBI, BRFU, BRBS and BRAS scales, univariate logistic models were used, in which the effect of these variables was measured by Odds Ratio (OR) [18]. The scale of continuous co-variates in the logit was evaluated. Residual analysis was performed in order to find outliers and influential observations. The goodness of fit of this model was evaluated with the Cessie Van-Howelinden test.

For the correlations between the HADS-D values and those of the QLQ-C30 and QLQ-BR23 questionnaires, the Pearson correlation coefficient was obtained, whenever both could be assumed to have come from populations with normal distribution, and the Spearman correlation coefficient was used in the other cases.

To compare the results of the EORTC QLQ-C30 and EORTC QLQ-BR23 subscales between the groups of patients with a major depressive episode, with depressive symptoms and without depressive symptoms one-way analysis of variance (ANOVA) was used when we could assume normality and homogeneity of the variances, followed by Tukey multiple comparisons test when a significant difference between the groups was present. In the cases were normality could not be assumed Kruskal–Wallis nonparametric test was used since in all the cases when the form of the distribution of those groups was similar. When a significant difference between those groups was present, the Dunn test with *p*-values adjusted with Holm correction was used.

The statistical analysis of the data was made with the statistical program R Project version 3.3 [19].

## Results

The study included 45 female patients. All the patients were evaluated with HADS and those with a positive score for depressive symptoms (HADS-D ≥ 8) were referenced to the Department of Psychiatry and Mental Health of our Hospital for further assessment and follow-up when required. 16 (35.6%) patients had a positive HADS-D questionnaire, of those, 7 (15,6%) patients had a major depressive episode confirmed by a structured psychiatric interview (6 patients had a major depressive disorder, recurrent episode and 1 had the diagnosis of major depressive disorder, single episode).

The characteristics of the patients, for several sociodemographic and clinical variables, are described in Table 1.

Chemotherapy regimens without monoclonal antibodies: doxorubicin + cyclophosphamide + docetaxel (*N* = 3); docetaxel monotherapy (*N* = 17); paclitaxel monotherapy (*N* = 2); carboplatin + gemcitabine (*N* = 2); carboplatin + paclitaxel (*N* = 2); gemcitabine + nab-paclitaxel (*N* = 1).

Treatment regimens with monoclonal antibodies: doxorubicin + cyclophosphamide + docetaxel + trastuzumab (*N* = 2); trastuzumab monotherapy (*N* = 7); docetaxel + trastuzumab + pertuzumab (*N* = 2); capecitabine + trastuzumab (*N* = 1); TDM-1 (*N* = 1); paclitaxel + trastuzumab (*N* = 1); docetaxel + trastuzumab (*N* = 4).

The mean values on the HADS-D questionnaire were 11.3 ± 2.5 for the group with depressive symptoms and 3.8 ± 2.1 (*p* < 0.001) for the group without depressive symptoms.

The association between having depressive symptoms, or not, and each of the sociodemographic and clinical variables showed to be significant or marginally significant in the following variables: stage of the tumour (*p* = 0.057), presence of distant metastasis (*p* = 0.072) and previous diagnosis of depression (*p* = 0.011).

For the variable stage of the tumour, it was particularly relevant to be in stage IV when compared to stage I (*p* = 0.028), since the odds of having depressive symptoms is about 9 times [IC_95%_ (OR) = (1.4; 82.1)] higher in the patients in stage IV.

In the patients with distant metastasis, the odds of depressive symptoms increased in almost four times [IC_90%_ (OR) = (1.1; 13.5)] when compared to those who did not have metastasis.

The patients previously diagnosed with depression before having breast cancer were eight times [IC_95%_ (OR) = (1.6; 62.0)] more likely to have depressive symptoms than those who were not.

**Figure 1.**
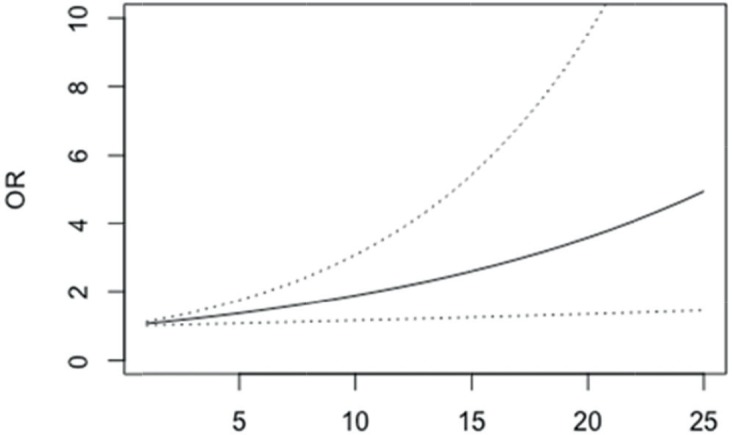
OR of not having depression relative to having depression in patients with differences in QOL scores between 1 and 25 points (95% confidence bands for the OR are shown in dash lines).

The overall QLQ-C30 score was also associated with the presence, or not, of depressive symptoms (*p* = 0.002). It can be noted that the odds of not having depressive symptoms are much higher when the difference in overall score increases. For example, a patient with 10 points more than another in the overall score has almost the double of the odds of not having depressive symptoms (Figure 1, Appendix).

When the two groups of patients were compared in the various functional scales of the QLQ-C30 questionnaire (Table 2), the only one in which there were no significant differences was for the subscale that evaluates the social functioning. In the other subscales, the mean values were higher in the group of patients without depressive symptoms, indicating that these patients have a better quality of life. The same conclusion is obtained for the global health status. Regarding the symptom scales, significant differences were found in the subscales fatigue (*p* = 0.024), pain (*p* = 0.037) and dyspnea (*p* = 0.009), being the mean values higher in the group of patients with depressive symptoms indicating a worse QoL in this group.

**Figure 2.**
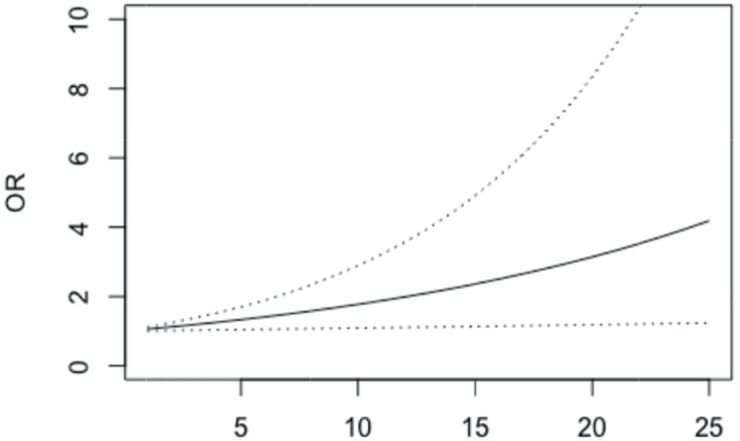
OR of not having depression in patients with differences in the BRBI scale scores between 1 and 25 points (95% confidence bands for the OR are shown in dash lines).

Similar interpretations of the overall QLQ-C30 score were observed with the body image scale (BRBI), *p* < 0.001 (Figure 2, Appendix).

**Figure 3.**
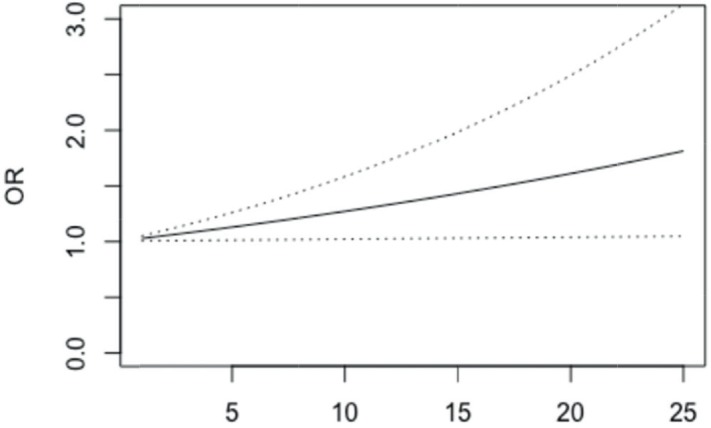
OR of not having depression in patients with differences in BRFU scale scores between 1 and 25 points (95% confidence bands for the OR are shown in dash lines).

There was also a significant association of depressive symptoms with the future perspectives (BRFU) scale, *p* = 0.022. The OR is bigger the greater the difference between the value in the BRFU scale. A patient with 10 points more than another in the BRFU scale is 27% more likely of not having depressive symptoms and if he has 25 points more, the odds of not having depressive symptoms are 81% higher (Figure 3, Appendix).

**Figure 4.**
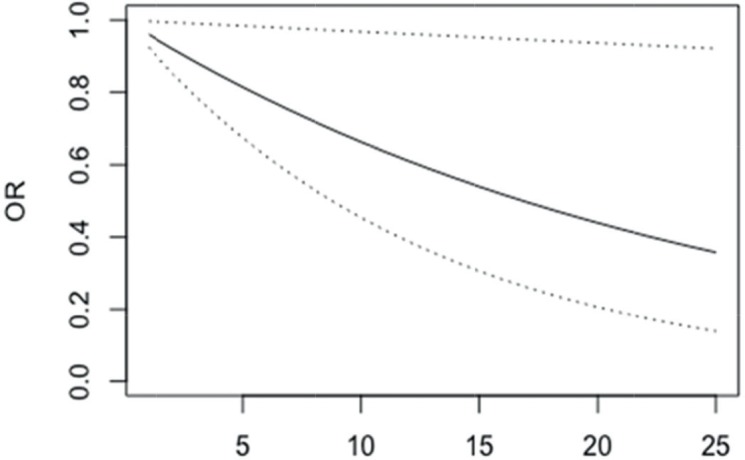
OR of not having depression in patients with differences in the BRBS scale scores between 1 and 25 points (95% confidence bands for the OR are shown in dash lines).

The presence of depressive symptoms is significantly associated with the score on the breast symptoms (BRBS) scale, *p* = 0.011. However, in this case, the OR of not having depressive symptoms decreases with higher scores on BRBS scale (Figure 4, Appendix). A patient with 10 more points in the BRBS score is 34% less likely to not having depressive symptoms and if the difference is 25 points, the odds of not having depressive symptoms are 66% lower.

**Figure 5.**
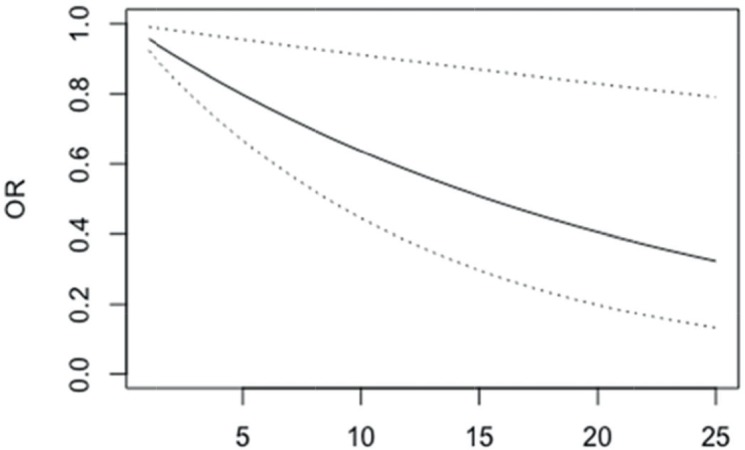
OR of not having depression in patients with differences in BRAS scale scores between 1 and 25 points (95% confidence bands for the OR are shown in dash lines).

Similar values and interpretations were observed in the arm symptom scale (BRAS), *p* = 0.005 (Figure 5, Appendix). When comparing the two groups of patients in the various functional subscales of the QLQ-BR23 (Table 3), no significant differences in the subscales that evaluate the sexual functioning and sexual pleasure were observed. In the other subscales, the mean values were higher in the group without depressive symptoms. In the symptom scales, the *p*-value was slightly higher than 5%, only in the subscales upset by hair loss and systemic therapy side effects. The mean values were higher in the group of patients with depressive symptoms indicating a worse QoL in this group.

Table 4 shows the OR and the 95% confidence interval of not having depressive symptoms when the scores of the scales increase 5, 10, 15 and 20 points. The role of metastatic disease as the confounder was examined but was only significant for BRAS (*p* = 0.038), and since the OR obtained were similar, we present in this case the OR estimated, based on the model without metastatic disease as a covariate.

Further analysis was done to compare the differences in the EORTC QLQ-C30 subscales between the groups of patients with a major depressive episode, with depressive symptoms and without depressive symptoms (Table 1, Appendix). Significant differences were observed between the group of patients with a major depressive episode and the group without depressive symptoms in the subscales PF (*p* = 0.003), RF (*p* = 0.041), CF (*p* = 0.007), FA (*p* = 0.010), PA (*p* = 0.042), QoL (*p* = 0.006). Also, significant differences were observed between the group of patients with a major depressive episode and with depressive symptoms when compared with the group without depressive symptoms in the DY (*p* = 0.031) and DI (*p* = 0.039) subscales. A significant difference was observed between the three groups in the EF subscale (*p* < 0.001) These results indicate a worse QoL in the group of patients with a major depressive episode and depressive symptoms. No significant differences were observed between the group of patients with a major depressive episode and the group of patients with depressive symptoms.

Another comparison was done between the groups of patients with a major depressive episode, depressive symptoms and without depressive symptoms for the EORTC QLQ-BR23 subscales (Table 2, Appendix). Significant differences were observed between the groups with a major depressive episode and depressive symptoms and the group without depressive symptoms in the subscales BRBI (*p* ≤ 0.001), BRBS (*p* = 0.007) and BRAS (*p* = 0.023) indicating a worse QoL in the group of patients with a major depressive episode and depressive symptoms. No significant differences were observed between the group of patients with a major depressive episode and the group of patients with depressive symptoms.

An analysis of the EORTC QLQ-C30 and QLQ-BR23 for the patients treated with chemotherapy regimens without monoclonal antibodies and those treated with treatment regimens with monoclonal antibodies, without considering the presence, or not, of depressive symptoms was done (Table 5). The mean values for each subscale of the QLQ-C30 questionnaire were found to be significant at 5% for the subscales RF, FA and DI and marginally significant at 10% for the subscales PF, EF, SF, PA and QoL. Those results indicated the healthiest level in these subscales in the group of patients treated with treatment regimens with monoclonal antibodies when compared to the group of patients treated with chemotherapy regimens without monoclonal antibodies. The patients treated with treatment regimens with monoclonal antibodies had more diarrhoea.

There were no significant differences between the types of treatments for the subscales of the QLQ-BR23 questionnaire (Table 3, Appendix).

An analysis was performed to compare the differences in the EORTC QLQ-C30 subscales between the patients with depressive symptoms treated with chemotherapy regimens without monoclonal antibodies and those treated with treatment regimens with monoclonal antibodies. The mean values for each subscale of the QLQ-C30 questionnaire that were found to be significant at 5% (FA) or marginally significant at 10% (EF and SF) are showed in Table 6. For the EORTC QLQ-BR23, only a significant difference at 5% was observed in the BRAS subscale (Table 7).

These results showed that the patients with depressive symptoms treated with regimens with monoclonal antibodies presented the healthiest level in these subscales when compared to the group of patients treated with chemotherapy regimens without monoclonal antibodies.

Another analysis was done to compare the EORTC QLQ-C30 subscales between the patients without depressive symptoms treated with chemotherapy regimens without monoclonal antibodies and those treated with treatment regimens with monoclonal antibodies. The mean values for each subscale of the QLQ-C30 questionnaire that were found to be significant at 5% (RF, DI and QoL) or marginally significant at 10% (PF, EF and PA) and are showed in Table 8. For the EORTC QLQ-BR23, only a significant difference at 5% was observed in the BRBS subscale (Table 9).

These results showed that the patients without depressive symptoms, treated with treatment regimens, with monoclonal antibodies, presented a better level in those subscales and a better QoL when compared to the group of patients treated with chemotherapy regimens without monoclonal antibodies.

**Figure 6.**
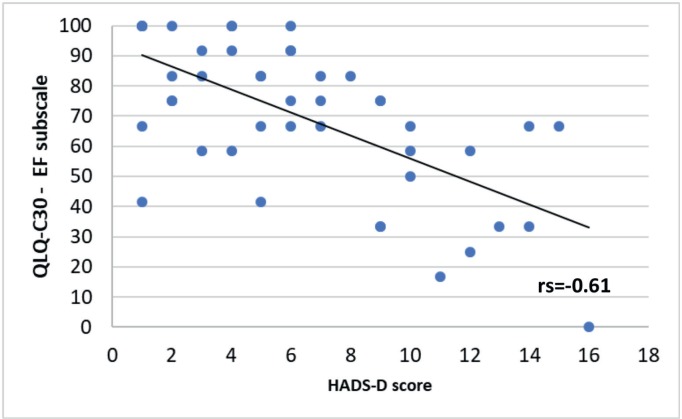
Correlation between HADS-D and the emotional functioning (EF) subscale of EORTC QLQ-C30.

**Figure 7.**
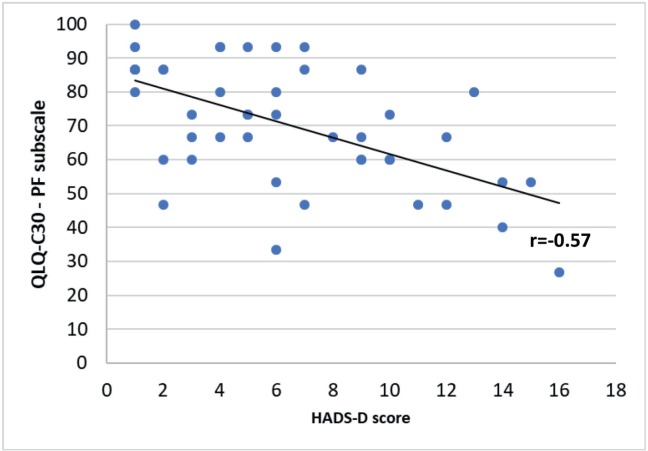
Correlation between HADS-D and the physical functioning (PF) subscale of EORTC QLQ-C30.

**Figure 8.**
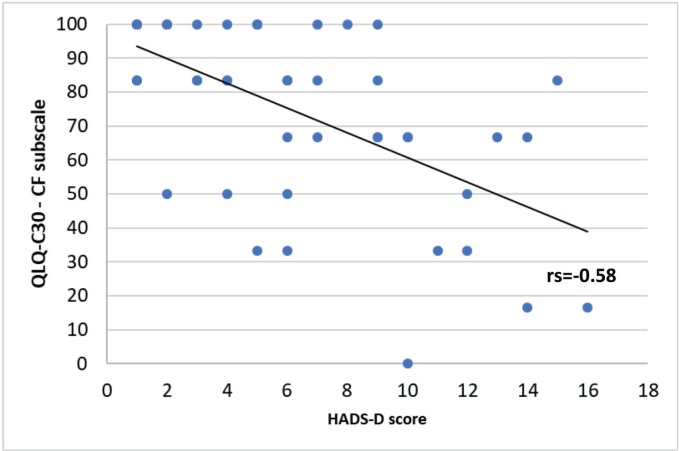
Correlation between HADS-D and the cognitive functioning (CF) subscale of EORTC QLQ-C30.

**Figure 9.**
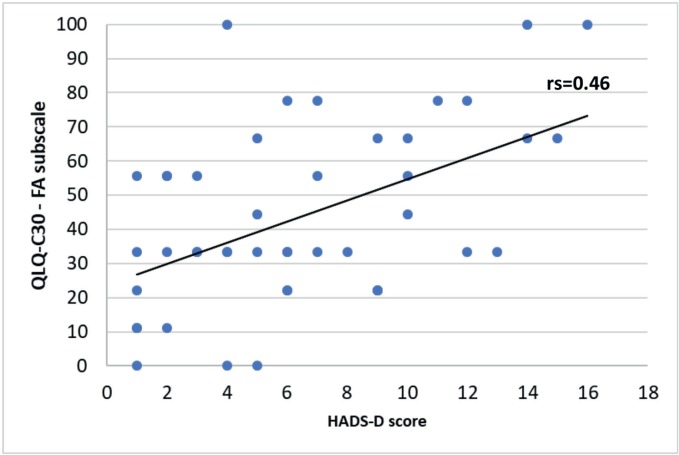
Correlation between HADS-D and the fatigue (FA) subscale of EORTC QLQ-C30.

**Figure 10.**
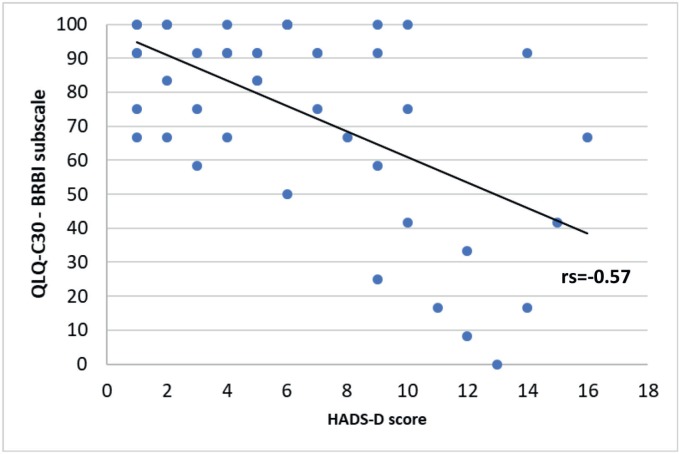
Correlation between HADS-D and the body image (BRBI) subscale of EORTC QLQ-C30.

A significant negative correlation was found between the HADS-D questionnaire and some of the QLQ-C30 questionnaire subscales. A Spearman correlation coefficient with an *r*_s_ = −0.61 for the emotional functioning (Figure 6, Appendix), a Pearson correlation coefficient an *r* = −0.57 for the physical functioning (Figure 7, Appendix), an *r*_s_ = −0.58 for the cognitive functioning (Figure 8, Appendix) and an *r*_s_ = −0.57 for the body image (Figure 10, Appendix) which translates a worse result in these subscales when the HADS-D questionnaire is higher. The fatigue symptoms subscale presented a positive Spearman correlation coefficient (*r*_s_ = 0.46), indicating an increase of this symptom in the patients with depressive symptoms (Figure 9 in Appendix).

## Discussion

In this study, 35.6% of the patients with breast cancer presented depressive symptoms and 15.6% with a major depressive episode. In the literature, the rate of depression in patients with breast cancer varies among the studies. In a study done by Derogatis *et al* [20] that included a majority of patients with breast cancer, 20% had major depressive episodes. A study of depression and anxiety among Malaysian breast cancer patients found that the rate of depression was 22% [21]. Another study involving Chinese patients with breast cancer observed a prevalence of depression of 57.9% [22].

The QoL was evaluated in a study with breast cancer patients and depression during adjuvant therapy. The authors concluded that the percentage of patients with anxiety or depression was higher in the chemotherapy group and that the patients with depression experienced overall poorer QoL [23].

Another study found that patients with stage IV breast cancer and those who had received chemotherapy also exhibited significantly lower QoL [24].

Purkayastha *et al* [25] observed that the group of patients with breast cancer and depression had lower scores in all the domains of QoL implying that the impairment in the physical health, psychological and social is associated with a higher psychological burden.

Studies evaluating the correlation of depression with disease progression in women with breast cancer showed inconsistent results [26]. A study found less depression in women with advanced breast cancer (4.5%) than in those with recurrent disease (15%) [27].

Pinder *et al* [28] found increased levels of depression in patients with breast cancer with the lowest socioeconomic status, the poorest performance status and at the end of life.

Another study comparing breast cancer versus benign tumour suggested that depression (*p* < 0.0001) and related symptoms, such as hopelessness (*p* < 0.001), loss of interest and pleasures (*p* < 0.001) could be considered risk factors for breast cancer.

Especially, during the first year after diagnosis, the prevalence of depression among women with early breast cancer is twice that in the general female population [30].

Some authors found that high levels of anxiety and depression were associated with higher mortality rates in cancer patients [31].

Depression is also linked to a reduced chance of survival in women with early-stage breast cancer [32] and also increased the risk of relapses following treatments [33].

Depression, but not anxiety, increased in the presence of distant metastases, relapse, or progression of the breast cancer [34].

Survival rates after a cancer diagnosis are reduced in people with pre-existing severe mental illness, with mortality risk 20% higher in patients with prior depression [35].

Major depression decreases motivation and reduces compliance with antineoplastic treatments, such as chemotherapy [36], and could also be an important predictor of late-stage breast cancer diagnosis [37] Reich *et al* [38] observed that major depression and depressive symptoms tend to be undertreated. This can be due to the fact that these patients are generally reluctant to reveal information regarding changes in their emotional states and also to the fact that some oncologists are not familiar with depressive symptoms screening.

73% of the depressed cancer patients do not receive effective psychiatric treatment, and only 5% see a mental health professional [39]. With our study, we tried to address this issue and provide better access to mental health care for the breast cancer patients examined.

Effective treatments of depression can lead to a reduction in depressive symptoms, improvement of psychosocial functioning, and greater QoL [40].

Therefore, the early detection of psychological symptoms and effective symptom management may maintain the effectiveness of the cancer treatments. This reinforces the need for a multidisciplinary approach concerning the treatment of breast cancer patients, namely, teams should incorporate psychologists and psychiatrists in order to provide early detection and treatment of depression.

In this study, the patients with breast cancer and depressive symptoms receiving chemotherapy and monoclonal antibodies showed a considerable decrease in the QoL when compared with the patients without depressive symptoms.

Through this data analysis, it was possible to observe that the presence of depressive symptoms was marginally significant in patients with advanced breast cancer when compared to the initial stages. The presence of distant metastasis and the previous diagnosis of depression are also possible factors that may increase the risk of depressive symptoms after the diagnosis of breast cancer.

There was also a correlation between a positive screening in the HADS-D questionnaire and the emotional, physical, cognitive functioning and the fatigue subscales of the QLQ-C30 questionnaire, with a worse result in the patients with depressive symptoms.

Regarding the QLQ-BR23 questionnaire, the only functional subscales in which there were no significant differences between the two groups were in the subscales that evaluate the sexual functioning and the sexual pleasure. For all the other subscales, the results were worse in the group of patients with depressive symptoms. In the symptom scales, the only subscales in which no differences were found between the two groups were upset by hair loss and systemic therapy side effects. All the other results of the subscales were worse in the group of patients with depressive symptoms.

As described in the previous results, it was also observed that the patients treated with treatment regimens containing monoclonal antibodies presented better results in various subscales of the EORTC QLQ-C30 and QLQ-BR23 than those who were treated with chemotherapy regimens without monoclonal antibodies without considering the presence or not of depressive symptoms. When considering the presence, or not, of depressive symptoms, the results in various subscales of the EORTC QLQ-C30 and QLQ-BR23 was also better in the patients treated with treatment regimens containing monoclonal antibodies when compared with the group treated with chemotherapy regimens without monoclonal antibodies.

## Conclusions

Despite the small sample of our groups, this study provided evidence that depressive symptoms in patients with breast cancer undergoing chemotherapy and monoclonal antibodies treatments detrimentally affected various aspects of the QoL in a real-world setting.

The development of new cancer treatments should consider the relevance of providing a higher QoL among cancer patients. Unfortunately, advances in therapy sometimes come with long-standing effects in the QoL.

Depression may reduce the efficacy of chemotherapy, and therefore the survival chances of cancer patients. Breast cancer is often accompanied by severe psychological distress, which is associated with a significant prevalence of anxiety and depression disorders in the course of the disease.

As our study was developed in a real-world setting, our results highlight that in order to improve the QoL of the patients with breast cancer, it is important to estimate the psychological burden caused by that type of cancer and its treatment and the need to find measures to reduce it. Patients with depressive symptoms should receive appropriate psychopharmacological treatment and support in order to improve their QoL.

Health professionals should be trained to intervene adequately with the patient and the family. For this, communication between oncologists and mental health teams is fundamental for the provision of a higher quality of care.

## Conflicts of interest

The authors have no conflicts of interest to declare.

## Funding statement

The authors received no financial support for the research, authorship, and/or publication of this article.

## Authors’ contributions

All the authors contributed to the elaboration of this paper and agreed with is content.

## Abbreviations

QoL: quality of life

HADS-D: Hospital Anxiety and Depression Scale

QL2: global health status and quality of life

FA: fatigue

NV: nausea and vomiting

PA: pain

DY: dyspnea

SL: insomnia

AP: appetite loss

CO: constipation

DI: diarrhoea

FI: financial difficulties

BRBI: composed by body image

BRSEF: sexual functioning

BRSEE: sexual enjoyment

BRFU: future perspective

BRST: systemic therapy side effects

BRBS: breast symptoms

BRAS: arm symptoms

BRHL: upset by hair loss

OR: Odds Ratio

## Figures and Tables

**Table 1.** Sociodemographic and clinical characteristics of the patients included in the study (mean age ± standard deviation).

		Group with depressive symptoms (*n*= 16)	Group without depressive symptoms (*n*= 29)	Total
Age	Years	56.5 ± 14.0	53.0 ± 11.0	54.3 ± 12.1
Marital status	Married / Civil union	8 (50.0%)	21 (72.4%)	29 (64.4%)
	Divorced	4 (25.0%)	4 (13.8%)	8 (17.8%)
	Single/Widowed	4 (25.0%)	4 (13.8%)	8 (17.8%)
Education level	Elementary school	6 (37.5%)	10 (34.5%)	16 (35.6%)
	Middle school	6 (37.5%)	6 (20.7%)	12 (26.7%)
	High school	2 (12.5%)	9 (31.0%)	11 (24.4%)
	Higher education	3 (12.5%)	4 (13.8%)	6 (13.3%)
Stage of the tumour	IA / IB	2 (12.5%)	12 (41.4%)	14 (31.1%)
	IIA / IIB	7 (43.8%)	8 (27.6%)	15 (33.3%)
	IIIA / IIIB / IIIC	1 (6.3%)	5 (17.2%)	6 (13.3%)
	IV	6 (37.5%)	4 (13.8%)	10 (22.2%)
Surgery	Not operated	3 (18.8%)	6 (20.7%)	9 (20.0%)
	Mastectomy	3 (18.8%)	5 (17.2%)	8 (17.8%)
	Lumpectomy	10 (62.5%)	18 (62.1%)	28 (64.4%)
Presence of metastasis	No	6 (37.5%)	4 (13.8%)	10 (22.2%)
	Yes	10 (62.5%)	25 (86.2%)	35 (77.8%)
Molecular subtype of breast cancer	Luminal A / Luminal B HER2 negative-like	5 (31.3%)	13 (44.8%)	18 (40.0%)
	Luminal B HER2 positive-like / HER2-type	9 (56.3%)	12 (41.4%)	21 (46.7%)
	Triple negative	2 (12.5%)	4 (13.8%)	6 (13.3%)
Medical treatment	Chemotherapy regimens without monoclonal antibodies	9 (56.3%)	18 (62.1%)	27 (60.0%)
	Treatment regimens with monoclonal antibodies	7 (43.7%)	11 (37.9%)	18 (40.0%)
	Adjuvant therapy	8 (50.0%)	20 (69.0%)	28 (62.2%)
	Neoadjuvant therapy	2 (12.5%)	5 (17.2%)	7 (15.6%)
	Palliative therapy	6 (37.5%)	4 (13.8%)	10 (22.2%)
Prior episode of depression	No	10 (62.5%)	27 (93.1%)	37 (82.2%)
	Yes	6 (37.5%)	2 (6.9%)	8 (17.8%)

**Table 2.** Mean values ± standard deviation of the EORTC QLQ-C30 subscales for the group of patients with depressive symptoms and the group of patients without depressive symptoms and *p*-values.

EORTC QLQ-C30	Group with depressive symptoms (*n*= 16)	Group without depressive symptoms (*n*= 29)	*p*-value
Functional scales			
Physical functioning (PF)	59.2 ± 15.0	76.3 ± 16.9	0.002^b^
Role functioning (RF)	52.1 ± 25.0	70.7 ± 29.8	0.019^b^
Emotional functioning (EF)	48.4 ± 24.0	81.0 ± 17.4	<0.001^b^
Cognitive functioning (CF)	57.3 ± 29.8	82.8 ± 21.1	0.003^b^
Social functioning (SF)	63.5 ± 35.1	81.6 ± 19.1	0.115^b^
Symptom scales/items			
Fatigue (FA)	55.6 ± 26.3	37.2 ± 24.5	0.024^a^
Nausea and vomiting (NV)	9.4 ± 18.2	7.5 ± 15.2	1.000^b^
Pain (PA)	47.9 ± 33.3	27.0 ± 23.3	0.037^b^
Dyspnea (DY)	20.8 ± 29.5	3.4 ± 10.3	0.009^b^
Insomnia (SL)	37.5 ± 29.5	25.3 ± 26.2	0.157^b^
Appetite loss (AP)	25.0 ± 33.3	11.5 ± 22.3	0.172^b^
Constipation (CO)	14.6 ± 29.7	5.7 ± 15.6	0.319^b^
Diarrhoea (DI)	14.6 ± 17.1	10.3 ± 23.3	0.150^b^
Financial difficulties (FI)	33.3 ± 34.4	31.0 ± 35.6	0.753^b^
Global health status (QoL)	43.3 ± 15.5	62.9 ± 19.6	0.002^b^

a *t*-test

b Wilcoxon–Mann–Whitney test

**Table 3.** Mean values ± standard deviation of the EORTC QLQ-B23 subscales for the group of patients with depressive symptoms and the group of patients without depressive symptoms and *p*-values.

QLQ-BR23	Group with depressive symptoms (*n*= 16)	Group without depressive symptoms (*n*= 29)	*p*-value
Functional scales			
Body image (BRBI)	52.1 ± 33.8	86.5 ± 14.2	0.001^b^
Sexual functioning (BRSEF)	13.5 ± 15.2	20.1 ± 19.1	0.289^b^
Sexual enjoyment (BRSEE)	28.6 ± 12.6	40.0 ± 22.5	0.216^b^
Future perspective (BRFU)	18.8 ± 27.1	41.4 ± 34.1	0.031^b^
Symptom scales / items			
Systemic therapy side effects (BRST)	44.9 ± 20.0	34.0 ± 22.4	0.072^a^
Breast symptoms (BRBS)			
Arm symptoms (BRAS)	28.1 ± 22.9	11.2 ± 19.2	0.002^b^
Upset by hair loss (BRHL)	34.0 ± 18.5	16.1 ± 18.0	0.006^b^
	48.9 ± 39.6	25.0 ± 31.5	0.057^b^

a *t*-test

b Wilcoxon–Mann–Whitney test

**Table 4.** OR and 95% profile likelihood confidence intervals of not having depressive symptoms between patients with differences in QOL, BRBI, BRFU, BRBS and BRAS scale scores of 5, 10, 15 and 20 points.

	Points of difference in the scale between the two groups of patients							
	5		10		15		20	
	OR	IC_95%_(OR)	OR	IC_95%_(OR)	OR	IC_95%_(OR)	OR	IC_95%_(OR)
Global health status (QOL)	1.4	(1.1; 1.8)	1.9	(1.2; 3.1)	2.6	(1.3; 5.4)	3.6	(1.4; 9.5)
Body image (BRBI)	1.3	(1.1; 1.6)	1.8	(1.2; 2.5)	2.4	(1.4; 4.0)	3.1	(1.5; 6.4)
Future perspective (BRFU)	1.1	(1.0; 1.3)	1.3	(1.0; 1.6)	1.4	(1.0; 2.0)	1.6	(1.0; 2.5)
Breast symptoms (BRBS)	0.82	(0.67; 0.98)	0.66	(0.45; 0.97)	0.54	(0.31; 0.95)	0.44	(0.21;0.94)
Arm symptoms (BRAS)	0.80	(0.67; 0.95)	0.64	(0.45; 0.91)	0.51	(0.30; 0.87)	0.41	(0.20;0.83)

**Table 5.** Mean ± standard deviation of EORTC subscales QLQ-C30 for the group of patients treated with chemotherapy regimens without monoclonal antibodies and patients treated with treatment regimens with monoclonal antibodies and *p*-values without considering the presence, or not, of depressive symptoms.

EORTC QLQ-C30	Chemotherapy regimens without monoclonal antibodies	Treatment regimens with monoclonal antibodies	p-value
Functional scales			
Physical functioning (PF)	66.4 ± 18.6	75.9 ± 16.0	0.084^a^
Role functioning (RF)	56.2 ± 29.2	75.9 ± 25.7	0.023^b^
Emotional functioning (EF)	64.5 ± 25.6	76.9 ± 23.5	0.097^b^
Social functioning (SF)	68.5 ± 29.7	85.2 ± 18.9	0.051^b^
Symptom scales / items			
Fatigue(FA)	51.0 ± 26.7	32.7 ± 22.4	0.021^a^
Pain (PA)	40.7 ± 30.8	25.0 ± 23.0	0.092^b^
Diarrhoea (DI)	6.2 ± 16.1	20.4 ± 25.9	0.023^b^
Global health status (QoL)	52.5 ± 17.6	63.0 ± 22.2	0.080^b^

a *t*-test

b Wilcoxon–Mann–Whitney test

**Table 6.** Mean ± standard deviation of EORTC subscales QLQ-C30 for the group of patients treated with chemotherapy regimens without monoclonal antibodies and patients treated with treatment regimens with monoclonal antibodies and *p*-values, in both groups with depressive symptoms.

EORTC QLQ-C30	Chemotherapy regimens without monoclonal antibodies	Treatment regimens with monoclonal antibodies	*p*-value
Functional scales			
Physical functioning (PF)	55.6 ± 15.6	63.8 ± 13.8	0.289^a^
Role functioning (RF)	44.4 ± 27.6	61.9 ± 18.5	0.174^a^
Emotional functioning (EF)	38.9 ± 23.2	60.7 ± 20.2	0.069^a^
Social functioning (SF)	50.0 ± 37.3	81.0 ± 24.4	0.079^a^
Symptom scales / items			
Fatigue(FA)	69.2 ± 22.8	38.1 ± 20.1	0.018^b^
Pain (PA)	57.4 ± 39.2	35.7 ± 20.2	0.206^a^
Diarrhea (DI)	11.1 ± 16.7	19.0 ± 17.8	0.389^b^
Global health status (QoL)	43.5 ± 10.8	47.6 ± 20.8	0.617

a *t*-test

b Wilcoxon–Mann–Whitney test

**Table 7.** Mean ± standard deviation of EORTC subscales QLQ-BR23 for the group of patients treated with chemotherapy regimens without monoclonal antibodies and patients treated with treatment regimens with monoclonal antibodies and *p*-values, both groups with depressive symptoms.

QLQ-BR23	Chemotherapy regimens without monoclonal antibodies	Treatment regimens with monoclonal antibodies	*p*-value
Functional scales			
Body image (BRBI)	40.7 ± 34.0	66.7 ± 29.7	0.132^a^
Sexual functioning (BRSEF)	13.0 ± 16.2	14.3 ± 15.0	0.863^b^
Sexual enjoyment (BRSEE)	25.0 ± 16.7	33.3 ± 0.0	0.564^b^
Future perspective (BRFU)	14.8 ± 24.2	23.8 ± 31.7	0.624^b^
Symptom scales / items			
Systemic therapy side effects (BRST)	43.4 ± 19.1	46.9 ± 22.4	0.737^a^
Breast symptoms (BRBS)	25.9 ± 18.8	31.0 ± 28.8	0.679^a^
Arm symptoms (BRAS)	43.2 ± 25.7	22.2 ± 9.1	0.045^a^
Upset by hair loss (BRHL)	45.8 ± 46.9	52.4 ± 32.5	0.765^b^

a *t*-test

b Wilcoxon–Mann–Whitney test

**Table 8.** Mean ± standard deviation of EORTC subscales QLQ-C30 for the group of patients treated with chemotherapy regimens without monoclonal antibodies and patients performing treatment regimens with monoclonal antibodies and *p*-values, both groups without depression.

EORTC QLQ-C30	Chemotherapy regimens without monoclonal antibodies	Treatment regimens with monoclonal antibodies	*p*-value
Functional scales			
Physical functioning (PF)	71.8 ± 17.9	83.6 ± 12.4	0.067^a^
Role functioning (RF)	62.0 ± 29.0	84.8 ± 26.3	0.012^b^
Emotional functioning (EF)	77.3 ± 15.1	87.1 ± 19.8	0.061^b^
Social functioning (SF)	77.8 ± 20.6	87.9 ± 15.1	0.201^b^
Symptom scales / items			
Fatigue(FA)	42.0 ± 24.3	29.3 ± 24.0	0.259^b^
Pain (PA)	32.4 ± 22.5	18.2 ± 22.9	0.071^b^
Diarrhoea (DI)	3.7 ± 15.7	21.2 ± 30.8	0.043^b^
Global health status (QoL)	56.9 ± 18.8	72.7 ± 17.5	0.020^b^

a *t*-test

b Wilcoxon–Mann–Whitney test

**Table 9.** Mean ± standard deviation of EORTC subscales QLQ-BR23 for the group of patients treated with chemotherapy regimens without monoclonal antibodies and patients performing treatment regimens with monoclonal antibodies and *p*-values, both groups without depression.

QLQ-BR23	Chemotherapy regimens without monoclonal antibodies	Treatment regimens with monoclonal antibodies	*p*-value
Functional scales			
Body image (BRBI)	85.2 ± 15.8	88.6 ± 11.3	0.727^b^
Sexual functioning (BRSEF)	20.4 ± 16.7	10.7 ± 23.3	0.740^b^
Sexual enjoyment (BRSEE)	36.4 ± 23.4	60.0 ± 19.2	0.347^b^
Future perspective (BRFU)	44.4 ± 34.3	36.4 ± 34.8	0.541^b^
Symptom scales / items			
Systemic therapy side effects (BRST)	33.1 ± 17.3	35.5 ± 21.1	0.729^a^
Breast symptoms (BRBS)			
Arm symptoms (BRAS)	15.7 ± 23.0	23.0 ± 5.7	0.029^b^
Upset by hair loss (BRHL)	18.5 ± 18.3	12.1 ± 17.5	0.313^b^
	21.4 ± 28.1	30.0 ± 36.7	0.678^b^

a *t*-test

b Wilcoxon–Mann–Whitney test

## Appendix

**Table 1.** Mean values ± standard deviation of the EORTC QLQ-C30 subscales for the group of patients with major depressive episode, group of patients with depressive symptoms and group of patients without depressive symptoms and *p*-values. Subscript with non-common letters means that the difference between the two groups is statistically significant.

EORTC QLQ-C30	Group with major depressive episode (*n*= 7)	Group with depressive symptoms (*n*= 9)	Group without depressive symptoms (*n*= 29)	*p*-value
Functional scales				
Physical functioning (PF)	53.3 ± 18.5^a^	63.7 ± 10.6^ab^	76.3 ± 16.9^b^	0.003 ^AN^
Role functioning (RF)	45.2 ± 24.9^a^	57.4 ± 25.2^ab^	70.7 ± 29.8^b^	0.041^KW^
Emotional functioning (EF)	39.3 ± 25.8^a^	55.5 ± 21.2^a^	81.0 ± 17.4^b^	<0.001^AN^
Cognitive functioning (CF)	50.0 ± 27.2^a^	63.0 ± 32.0^ab^	82.8 ± 21.1^b^	0.007^KW^
Social functioning (SF)	61.9 ± 40.5^a^	64.8 ± 32.7^a^	81.6 ± 19.1^a^	0.095 ^AN^
Symptom scales / items				
Fatigue (FA)	69.8 ± 25.4^a^	44.4 ± 22.2^ab^	37.2 ± 24.5^b^	0.010 ^AN^
Nausea and vomiting (NV)	9.5 ± 18.9^a^	9.3 ± 18.8^a^	7.5 ± 15.2^a^	0.983^KW^
Pain (PA)	54.8 ± 41.6^a^	42.6 ± 26.5^ab^	27.0 ± 23.3^b^	0.042^KW^
Dyspnea (DY)	19.1 ± 26.2^ab^	22.2 ± 33.3^b^	3.4 ± 10.3^a^	0.031^KW^
Insomnia (SL)	33.3 ± 38.5^a^	40.7 ± 22.2^a^	25.3 ± 26.2^a^	0.234^KW^
Appetite loss (AP)	38.1 ± 40.5^a^	14.8 ± 24.2^a^	11.5 ± 22.3^a^	0.165^KW^
Constipation (CO)	23.8 ± 37.1^a^	7.4 ± 22.2^a^	5.7 ± 15.6^a^	0.169^KW^
Diarrhoea (DI)	4.8 ± 12.6^ab^	22.2 ± 16.7^b^	10.3 ± 23.7^a^	0.039^KW^
Financial difficulties (FI)	23.8 ± 25.2^a^	40.7 ± 40.1^a^	31.0 ± 35.6^a^	0.708^KW^
Global health status (QoL)	38.1 ± 11.6^a^	50.9 ± 16.4^ab^	62.9 ± 19.6^b^	0.006 ^AN^

AN ANOVA test

KW Kruskal–Wallis test

**Table 2.** Mean values ± standard deviation of the EORTC QLQ-B23 subscales for the group of patients with major depression, group of patients with depression and group of patients without depression and *p*-values. Subscript with non-common letters means that the difference between the two groups is statistically significant.

EORTC QLQ-BR23	Group with major depressive episode (*n*= 7)	Group with depressive symptoms (*n*= 9)	Group without depressive symptoms (*n*= 29)	*p*-value
Functional scales				
Body image (BRBI)	44.0 ± 34.6^a^	58.3 ± 33.9^a^	86.5 ± 14.2^b^	<0.001^AN^
Sexual functioning (BRSEF)	7.2 ± 13.1^a^	18.5 ± 15.5^a^	20.1 ± 19.1^a^	0.216^KW^
Sexual enjoyment (BRSEE)	16.7 ± 23.6^a^	33.3 ± 0.0^a^	40.0 ± 22.6^a^	0.305^AN^
Future perspective (BRFU)	19.0 ± 26.2^a^	18.5 ± 29.4^a^	41.4 ± 34.1^a^	0.094^KW^
Symptom scales / items				
Systemic therapy side effects (BRST)	51.7 ± 25.3^a^	39.7 ± 14.1^ab^	34.0 ± 18.5^b^	0.092 ^AN^
Breast symptoms (BRBS)	23.8 ± 15.5^a^	31.5 ± 27.9^a^	11.2 ± 19.2^a^	0.007^KW^
Arm symptoms (BRAS)	38.1 ± 28.6^a^	30.9 ± 17.4^a^	16.1 ± 18.0^b^	0.023^KW^
Upset by hair loss (BRHL)	44.4 ± 45.5^a^	51.9 ± 37.7^a^	25.0 ± 31.5^a^	0.140^KW^

AN ANOVA test

KW Kruskal–Wallis test

**Table 3.** Mean ± standard deviation of EORTC subscales QLQ-BR23 for the group of patients performing chemotherapy regimens without trastuzumab and patients performing treatment regimens with trastuzumab and *p*-values without considering the presence, or not, of depressive symptoms.

EORTC QLQ-BR23	Chemotherapy regimens without trastuzumab	Treatment regimens with trastuzumab	*p*-value
Functional scales			
Body image (BRBI)	70.4 ± 31.2	80.1 ± 22.5	0.410^b^
Sexual functioning (BRSEF)	17.9 ± 16.6	17.6 ± 20.2	0.786^b^
Sexual enjoyment (BRSEE)	33.3 ± 21.8	42.9 ± 16.3	0.343^b^
Future perspective (BRFU)	34.6 ± 33.9	31.5 ± 33.3	0.778^b^
Symptom scales / items			
Systemic therapy side effects (BRST)	36.5 ± 18.3	39.9 ± 21.7	0.569^a^
Breast symptoms (BRBS)	19.1 ± 21.9	14.4 ± 22.3	0.183^b^
Arm symptoms (BRAS)	26.7 ± 23.7	16.0 ± 15.3	0.153^b^
Upset by hair loss (BRHL)	30.3 ± 37.0	39.2 ± 35.8	0.394^b^

a *t*-test

b Wilcoxon–Mann–Whitney test
